# Social Support, but Not Perceived Food Environment, Is Associated with Diet Quality in French-Speaking Canadians from the PREDISE Study

**DOI:** 10.3390/nu11123030

**Published:** 2019-12-12

**Authors:** Elise Carbonneau, Benoît Lamarche, Julie Robitaille, Véronique Provencher, Sophie Desroches, Marie-Claude Vohl, Catherine Bégin, Mathieu Bélanger, Charles Couillard, Luc Pelletier, Luigi Bouchard, Julie Houle, Marie-France Langlois, Louise Corneau, Simone Lemieux

**Affiliations:** 1 Centre Nutrition, santé et société (NUTRISS), Institute of Nutrition and Functional Foods, Université Laval, 2440 Hochelage Boulevard, QC G1V 0A6, Canada; 2 School of Nutrition, Université Laval, 2425, rue de l'Agriculture, QC G1V 0A6, Canada; 3 School of Psychology, Laval University, 2325 allée des bibliothèques, QC G1V 0A6, Canada; 4 Department of Family Medicine, Université de Sherbrooke, 18 avenue Antonine-Maillet, Moncton, NB E1A 3E9, Canada; 5 School of Psychology, University of Ottawa, 75 Laurier Ave E, Ottawa, ON K1N 6N5, Canada; 6 Department of Medical Biology, CIUSSS du Saguenay-Lac-St-Jean, Saguenay, QC G7H 5H6, Canada; 7 Department of Biochemistry, Université de Sherbrooke, 2500 Boulevard de l'Université, Sherbrooke, QC J1K 2R1, Canada; 8 Nursing Department, Université du Québec à Trois-Rivières, 3351 Boulevard des Forges, Trois-Rivières, QC G8Z 4M3, Canada; 9 Department of Medicine, Division of Endocrinology, Université de Sherbrooke, 3001 12e Ave N, Sherbrooke, QC J1H 5N4, Canada

**Keywords:** social support, food environment, diet quality, sociodemographic characteristics, healthy eating index

## Abstract

The objectives were to assess whether social support for healthy eating and perceived food environment are associated with diet quality, and to investigate if sociodemographic characteristics moderate these associations. A probability sample of French-speaking adults from the Province of Québec, Canada, was recruited in the context of the PREDISE study. Participants reported their perceptions of supportive and non-supportive actions related to healthy eating from close others at home and outside of home (*n* = 952), and of the accessibility to healthy foods (*n* = 1035). The Canadian Healthy Eating Index (C-HEI) was calculated based on three Web-based 24 h food recalls. Multiple linear regression models showed that supportive (B = 1.50 (95% CI 0.46, 2.54)) and non-supportive (B = −3.06 (95% CI −4.94, −1.18)) actions related to healthy eating from close others at home were positively and negatively associated with C-HEI, respectively, whereas actions from close others outside of home were not. The negative association between non-supportive actions occurring at home and C-HEI was stronger among participants with lower (vs. higher) levels of education (*p* interaction = 0.03). Perceived accessibility to healthy foods was not associated with C-HEI (*p* > 0.05). These results suggest that the social environment may have a stronger influence on healthy eating than the perceived physical environment. This adds support for healthy eating promotion programs involving entire families, especially for more socioeconomically disadvantaged individuals, whose efforts to eat healthily may be more easily thwarted by non-supportive households.

## 1. Introduction

A wide range of determinants have a potential influence on dietary intakes and eating behaviors [1]. It has been proposed that determinants of healthy behaviors can be differentiated into three broad interrelated categories, namely motivations, abilities, and opportunities [2,3]. While the first two categories are considered as personal or individual determinants, the third relates to the social and physical environments that contribute to opportunities for engaging in healthy behaviors. While various individual factors have been widely investigated in observational and intervention studies over the last decades, there is a growing scientific interest on determinants related to the social and physical environments that can influence food intake.

Although the literature has shown inconsistent evidence up until now [4,5,6,7], it has been suggested that social environment may have a more consistent influence on food behaviors than the physical environment [3]. Social environment includes factors such as being married, the household size, having children, as well as relational factors such as parental modeling, social isolation, and social support, the latter being the most frequently studied. Social support can be defined as “a transactional communicative process, including verbal and/or nonverbal communication, that aims to improve an individual’s feelings of coping, competence, belonging, and/or esteem” [8]. Most studies that have investigated the association between social factors and food intake to date have targeted specific subgroups of the population such as older adults [9,10,11], adolescents and children [12,13], or individuals affected by specific diseases [14,15]. Results showed that social factors such as social support and parental modeling are usually associated with better diet quality. Social support, has also been investigated in weight loss situations [16], and has been found to contribute to effective weight loss interventions [17]. However, less is known regarding the association between social environment and food intake in the general population [18,19]. Also, only few studies pertaining to social environment have evaluated food intake using proxies of overall diet quality [18,19,20], the majority having specifically studied the associations between social factors and either fruit and vegetable or fat intakes. Therefore, whether social support provided by family and friends towards healthy eating facilitates overall healthier eating habits remains uncertain.

Regarding the physical food environment, many authors have used store audits [21,22,23] or geocoding data (e.g., amount of food retailers in a given neighborhood, distance from participants’ home to groceries) [24,25,26,27] to evaluate how objectively measured features of the environment influence dietary intakes. These studies have come to conflicting results, which could be explained by consumers not always shopping at the food retailer closest to home [24,28] and by different individuals having different perceptions of the same food environment. In order to overcome these confounding factors, others have chosen to use subjective measures of the food environment, such as participants’ perceptions of the availability, accessibility or affordability of healthy and unhealthy foods in their neighborhood [29,30,31,32]. Although measures of the perceived availability of healthy foods are more consistently related to dietary outcomes [7], the use of non-validated tools in the vast majority of studies limits inferences that can be drawn from these studies. Also, many of these studies were performed in socioeconomically disadvantaged samples [23,29,30,33,34]. Therefore, less is known about the impact of the physical food environment among individuals drawn from the general population and with various socioeconomic status. The use of non-validated instruments for the measures of both the environment and food intakes was raised by Brug [3] who also pointed to the lack of consideration for potential covariates and moderators (such as sociodemographic characteristics) to better understand the associations between the social and physical food environment and diet quality in the literature.

In order to overcome these methodological issues, the present study was preceded by validation studies aimed at developing specific instruments for the measure of the perceived social [35] and physical food environment [36] as well as the assessment of dietary intakes [37,38], in a sample distinct from the present study but with similar characteristics. Using these validated tools, the objectives of this study were 1) to assess whether and how social support for healthy eating is associated with overall diet quality and to investigate if sociodemographic characteristics moderate these associations, and 2) to assess whether and how perceived food environment is associated with overall diet quality to investigate if sociodemographic characteristics moderate these associations, in a sample of French-speaking adults from the Province of Québec, Canada.

## 2. Materials and Methods 

### 2.1. Participants and Procedures

Participants were recruited as part of the PREDISE (PRÉDicteurs Individuels, Sociaux et Environnementaux) study, a multicentre cross-sectional study aimed at identifying determinants of the adherence to dietary guidelines among French-speaking adults from the Province of Québec, Canada. Recruitment and procedures were described previously [39]. Briefly, a probability sample of French-speaking men and women from the Province of Québec aged 18 to 65 were included in the study. They were recruited using random digit dialing in order to represent the French-speaking adult population of five regions of recruitment based on sex and age. Individuals had to have an Internet access for the completion of questionnaires. Exclusion criteria were pregnancy, lactation, and intestinal malabsorption. Once recruited, participants had three weeks to complete a series of online questionnaires on an Internet platform and they visited a research center affiliated to the PREDISE study in their region for anthropometric and blood pressure measurements, and for blood sampling.

**Figure 1:**
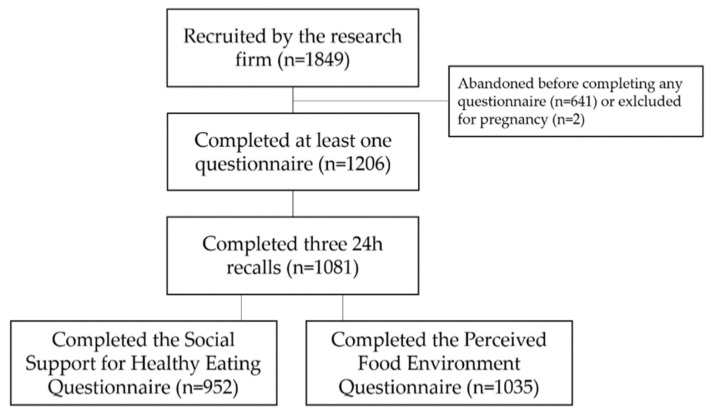
Flowchart of the PREDISE study.

On a total of 1849 individuals recruited, 1206 were included in the PREDISE project. A total of 1081 completed three 24 h recalls, among which 952 completed the Social Support for Healthy Eating Questionnaire and 1035 completed the Perceived Food Environment Questionnaire (see Figure 1 for the study flowchart and information on excluded participants).

### 2.2. Measures

Social support for healthy eating was assessed using the Social Support for Healthy Eating Questionnaire, a validated tool that was developed for the French-speaking adult population of the Province of Québec [35]. The questionnaire consists of 20 items in which participants are asked to rate how frequently, in the past month, close others had taken particular actions or said particular statements related to healthy and unhealthy eating in two different environments, i.e., at home (defined as “people living with you, e.g., family members, partner, roommate”) and outside of home (defined as “people with whom you share meals, but who do not live with you, e.g., friends, colleagues”). Twelve items relate to supportive actions (sample items include: “…proposed that we eat healthier” or “… gave me ideas to eat more healthy foods”) and eight items relate to non-supportive actions (sample items include: “…criticized the healthy foods that I served them” or “…said that healthy foods do not taste good”. Items were rated on a five-point Likert scale (“never”, “rarely”, “sometimes”, “often”, and “very often”). Answers were scored on four subscales for each participant, i.e., supportive actions at home; non-supportive actions at home; supportive actions outside of home; and non-supportive actions outside of home. Subscales scores were obtained by calculating the means of the items, with a maximum score of five. Higher scores for the supportive and the non-supportive scales mean a higher frequency of these types of action. All four subscales showed good internal consistency in the present sample with Cronbach α ranging from 0.76 to 0.90. 

Perceived food environment was assessed using the Perceived Food Environment Questionnaire, specifically developed and validated to assess perceived accessibility to healthy and unhealthy foods among French-speaking adults in the Province of Québec [36]. The tool is composed of two subscales, the first assessing accessibility to healthy foods through six items (e.g., “I consider that the quantity of healthy foods offered by my main food retailer is sufficient”) and the second including three items related to accessibility to unhealthy foods (e.g., “I consider that fast-food restaurants are easily accessible from my home”). The questionnaire contains items related to the food environment near home and workplace. Items are rated on a five-point scale, from “strongly disagree” to “strongly agree”, with the addition of a “not applicable” option for the items pertaining to the work environment. In the present sample, the subscale assessing accessibility to healthy foods showed adequate internal consistency (Cronbach α = 0.70), but the three items related to accessibility to unhealthy foods were not considered as internally reliable (Cronbach α = 0.49). It was therefore decided that the subscale assessing perceived accessibility to healthy foods would be the only one used in the present study. In the questionnaire, participants were also asked to report travel time (less than 10 min, 10–20 min, or more than 20 min) from home to the main food retailer by car and on foot.

It is important to specify that we decided not to provide participants with a precise definition of “healthy eating” and “unhealthy eating” to be sure not to influence their answers in the 24 h food recalls. However, in the Social Support for Healthy Eating questionnaire, “junk foods” was defined and examples of “healthy foods” were given to participants. In the Perceived Food Environment questionnaire, a brief definition of “healthy foods” was also presented.

Diet quality was assessed with the Canadian Healthy Eating Index 2007 (C-HEI). Participants completed three Web-based 24 h food recalls, using an application (R24W) developed by our research team [38]. Participants had to report all foods and drinks consumed from midnight to midnight on three days generated at random by the Web-based system. The R24W was validated for the French-speaking adult population of Province of Québec [37,40,41]. Using data generated by the R24W, participants’ overall diet quality was calculated through the C-HEI [42]. The index was developed to reflect 2007 Canada’s Food Guide recommendations for healthy eating [43]. The C-HEI is composed of eight adequacy components (total fruits and vegetables, whole fruits, dark-green and orange vegetables, grain products, whole-grain products, milk and alternatives, meat and alternatives, and unsaturated fat) and three moderation components (saturated fat, sodium, and “other foods” i.e., foods that are not part of those recommended by Canada’s Food Guide). Each component is evaluated on 5, 10, or 20 points, for a total maximum score of 100 (see full description of the C-HEI score in Supplementary Table S1). The C-HEI was computed as a continuous variable in the analyses based on the average intake of foods and nutrients from the three 24 h recalls [42].

In a sociodemographic questionnaire, participants reported their age, their highest level of education (i.e., no diploma, elementary school, high school, college, or university), and their annual household income. For the analyses, education was classified in two categories, i.e., high school or less vs. college or university, and income was divided in two categories, i.e., participants living under the low-income cut-off according to the Québec Institute of Statistics, based on the household size [44] vs. those living over the low-income cut-off. Participants also provided information on marital status, i.e., married (or living common-law) vs. other status, and living arrangement (i.e., living with partner and children, partner only, children only, other family member, roommate, or living alone). Smoking status was assessed, and participants were classified as current smokers (frequently or occasionally) vs. non-smokers/former smokers. Nutrition knowledge was also assessed [45]. 

### 2.3. Ethics

The PREDISE study was conducted according to the guidelines laid down in the Declaration of Helsinki. The research project received approval from the Research Ethics Committees of Université Laval (ethics number: 2014-271), Centre hospitalier universitaire de Sherbrooke (ethics number: MP-31-2015-997), Institut de recherches cliniques de Montréal (ethics number: 2015-02), and Université du Québec à Trois-Rivières (ethics number: 15-2009-07.13). All participants gave implied consent for the first phase of the study (i.e., completion of online questionnaires) and written informed consent for the second phase (i.e., anthropometric and blood pressure measurements and blood sampling at the research center). As a compensation for the first phase of the study, two iPads and 40 CAD 100 gift certificates were randomly drawn among participants who completed the online questionnaires. Participants also received a CAD 50 compensation at their visit to the research center.

### 2.4. Statistical Analyses

For the first objective of the study, participants included were those having completed all three 24 h recalls and having no missing data in the Social Support for Healthy Eating Questionnaire. Differences in social support according to sociodemographic characteristics were assessed using Student’s t-test and generalized linear models (GENMOD). Multiple linear regression analyses were performed to assess how social support was associated with the C-HEI, the proxy of diet quality (dependent variable). The four social support subscales (i.e., supportive actions at home, non-supportive actions at home, supportive actions outside of home, and non-supportive actions outside of home) were used as the independent variables. We also tested interaction terms to evaluate if some sociodemographic characteristics (i.e., sex, age, income, education, living with a partner vs. living with someone else, living alone vs. not living alone) moderate the association between social support and the diet quality. Stratified analyses were then performed according to significant moderators. For the second objective of the study, participants included were those having completed all three 24 h recalls, and having no missing data in the Perceived Food Environment Questionnaire. The same analyses as described for the first objective were used, and the independent variables in the regression models were the perceived accessibility to healthy foods and the travel time by car (travel time was dichotomised: 10 min or less vs. more than 10 min). The potential moderators tested were sex, age, income, and education. Covariates included in all models were sex, age, education, income, nutrition knowledge, marital status, and smoking status, and were all found to be significantly associated with diet quality in previous analyses [46]. Since misreporting of dietary intake can be an issue causing systematic bias, reporting status (i.e., under-reporter, plausible reporter, or over-reporter) was also included as a covariate. As previously detailed [47], the reporting status was assessed using the method by Huang et al., [48] according to which under- and over-reporters are those with a calculated energy intake: predicted energy requirement ratio <0.78 and  >1.22, respectively. Missing data for education, income, marital status, smoking status, and reporting status were imputed using the MI procedure. Less than 10% of participants had missing information, and missing data pattern was arbitrary, we therefore performed multivariate imputation using a fully conditional specification (FCS) logistic regression method for classification variables. The unstandardized betas are presented for the results of the regression analyses. Statistical tests were two-sided and differences or associations at *p* < 0.05 were considered significant. Analyses were performed using the SAS version 9.4 (SAS Institute Inc., Cary, NC, USA).

## 3. Results

### 3.1. Objective 1: Social Support for Healthy Eating

Characteristics of the 952 participants included for the first objective are presented in Table 1. 

The mean C-HEI was 57.1 ± 14.1 on a scale of 0 to 100 (note that scores from 50 to 80 can be categorized as a “diet that require improvements [42]”). Mean scores for the four subscales of the Social Support for Healthy Eating questionnaire are presented in Table 2. Overall, participants seemed to perceive more supportive actions than non-supportive ones from their close others both at home and outside of home. Women perceived less supportive actions and more non-supportive actions at home than men. However, women perceived more supportive actions outside of home than men (see Table 2). Age groups’ differences were also observed (see details in Table 2). Overall, older participants perceived less supportive and non-supportive actions from family and friends at home and outside of home. Social support at home also varied according to participants’ living arrangement (Table 2). Participants living either with a partner, with a partner and children, or with another family member were the ones who perceived the highest supportive actions at home. As expected, participants who reported living alone perceived less social support at home. As presented in Table 2, more supportive actions at home were reported by individuals with higher income, and more supportive actions outside of home were reported by individuals with higher income and education levels.

Unadjusted correlations between the four subscales of social support for healthy eating and C-HEI are presented in Supplementary Table S2. Both supportive actions both at home and outside of home were positively associated with C-HEI (*p* < 0.05).

Multiple linear regression analyses showed that supportive and non-supportive actions at home were respectively associated positively and negatively with C-HEI while neither type of actions outside of home was associated with C-HEI (Table 3). 

Sex, age, and annual household income were not found to significantly moderate the associations between social support subscales and C-HEI (*p* interaction > 0.05). However, education significantly moderated the association between non-supportive actions at home and C-HEI (*p* interaction = 0.03). Results of the multiple linear regression analyses stratified by education levels showed that the non-supportive actions at home were negatively associated with C-HEI in all participants, but the association was stronger among participants with a high school diploma or less (see Table 4). No significant interactions were found between social support subscales and the two variables related to living arrangement (*p* > 0.05).

### 3.2. Objective 2: Perceived Food Environment

Characteristics of the 1035 participants included for the second objective were similar to those of participants included for the first objective (see Supplementary Table S3). 

On a scale of one to five, the average accessibility to healthy foods score was 3.8 ± 0.5 (5.4% of participants had a score below three, 50.0% had a score between three and four, and 44.6% had a score of four or higher). As shown in Table 5, there were no differences in the score between men and women nor between age groups. The accessibility to healthy foods score was, however, lower for participants with lower annual household income and for participants with lower educational attainment. The accessibility to healthy foods score did not differ between the 5 recruitment regions (*p* = 0.26). Travel time from home to the main food retailer on foot was less than 10 min for 28.7% of the participants, 10 to 20 min for 28.2% of the participants, and more than 20 min for 43.1% of the participants. Travel time from home to the main food retailer by car was less than 10 min, 10 to 20 min, and more than 20 min for respectively 75.6%, 21.1%, and 3.4% of the participants.

As shown in Table 6, neither perceived accessibility to healthy foods score nor travel time by car was significantly associated with C-HEI. Results were similar when the variable “travel time on foot” was used instead of “travel time by car”. None of the sociodemographic characteristics tested (i.e., sex, age, income, and education) was found to moderate the association between the accessibility to healthy foods score and C-HEI (*p* interaction > 0.05).

## 4. Discussion

The present study aimed to explore the role of social and perceived physical food environment in the adherence to healthy eating recommendations in a probability sample of French-speaking adults from the Province of Québec. Representing about one fifth of the population of Canada, the French-speaking population of the Province of Québec has been found to differ from other Canadians with respect to food intakes and attitudes towards eating [49,50], stressing the relevance of studying determinants of healthy eating in this specific population.

To the best of our knowledge, the present study is one of the first to assess social support from two different sources (i.e., close others at home and outside of home) in association with a proxy of overall diet quality. Our results suggest that supportive and non-supportive actions from individuals with whom one lives have the potential of enhancing or thwarting the adherence to a healthy diet whereas supportive and non-supportive actions from individuals outside of home were not found to significantly influence diet quality. These results may be explained by the fact that many individuals share more meals with people they live with than with friends and coworkers [50]. In this regard, there may be more social interaction regarding food at home due to food-related tasks, such as food planning, procurement, and cooking, which can be shared with family members, partners, or roommates [51]. Furthermore, as we have previously proposed, there may be more stability in individuals with whom one shares meals at home than outside of home [35], which may offer more opportunities for influencing one’s opinion about healthy eating and intention to eat healthily in the home environment than outside of home. In other studies, family support, compared to support from friends, has been found to be more consistently associated with intake of fruit and vegetable and fast food, or with low-fat diets [52,53,54]. The type of support received from close others in different contexts can impact on the styles of motivation regulating one’s health-related behaviors. Indeed, the role of social support has been positively associated with autonomous motivation for behavioral change in interventions aiming at weight loss [16], tobacco cessation [55], and increased physical activity [56], but less is known regarding specifically the adherence to healthy eating recommendations [57]. Therefore, it can be hypothesized that motivational processes play a role in the association between social support and diet quality observed in the present study and this avenue should be further investigated.

There is a growing body of literature on the impact of social support on food intake, and more specifically on healthy eating. However, as raised by Brug [3] in a narrative review of systematic reviews on the topic, very few studies to date have assessed the influence of sociodemographic characteristics as potential moderators of the association between food environment and diet quality. Studying moderators of the associations between social/physical food environment and diet quality may be helpful for a better understanding of the conditions under which the food environment impacts food intake. Among the sociodemographic characteristics tested in the present study, education was found to be a significant moderator of the association between social support and diet quality. These results suggest that some individuals, namely those with lower education level, may be more vulnerable to non-supportive actions from their close others. These findings provide insights for the explanation of the well-documented differences in diet quality between socioeconomically advantaged and disadvantaged individuals [42,58,59,60]. Our results are in line with previous research [52] suggesting that healthy eating promotion programs involving entire families for an enhanced social support at home may help reduce the impact of the socioeconomic status on diet quality.

The nonsignificant interaction between sex and social support we noted suggests that men and women do not benefit differently from social support when it comes to overall diet quality, as other authors have previously reported [61]. However, gender differences have previously been observed. In fact, in a sample of US adults, the perception of a higher social support from close others was related to better dietary practices among women but not men [62]. It was also found that men benefited more from the support of their heterosexual partner than women in terms of dietary change intentions and dietary behavior (low-fat diet) [63]. 

Our results have shown that the perceived accessibility to healthy foods and the distance from home to the main food retailer are not significantly associated with the overall diet quality in our French-Canadian sample from the Province of Québec. Many other studies have found no significant association between food environment and food intake [24,31,34,64], although it has been suggested that associations between environment and behaviors are stronger when subjective (e.g., perception of the accessibility to healthy foods) rather than objective (e.g., store audits) measures are used [7,65,66]. In the present study, the absence of association may be due to the low variability in the independent variables studied. Indeed, less than 6% of the sample had a negative perception of the accessibility to healthy foods (i.e., mean score below three out of five; three representing a neutral opinion), and 44% had a mean score of four or higher, meaning that they agreed or strongly agreed with most of the items. More than 75% of the sample also reported that travel time by car from home to the main food retailer was less than 10 min. This low variability in the independent variables may be due to the study design, where participants had to visit one of the research centers for blood sampling as well as for measurements of anthropometric variables and blood pressure. Therefore, we may have recruited participants living near city centers where the accessibility to healthy foods is often higher. Different results may have been observed if we had recruited more participants living in rural areas. Based on the results we obtained, it can be hypothesized that the perception of physical food environment is less likely to have an impact on diet quality in urban areas, but more research is needed to further examine this hypothesis.

One of the objectives of the present study was to assess whether some sociodemographic characteristics moderated the association between perceived physical food environment and diet quality, or in other words, whether some subgroups of the population are more likely to be influenced by their perception of the physical environment when it comes to healthy eating. Such interactions have been rarely tested, and it has been previously pointed out as a major issue of studies interested in the association between environment and food intake [3]. A recent systematic review on socioeconomic differences in the association between the food environment and dietary behaviors concluded that there is no clear evidence of such differences [67]. Two studies have observed differences between ethnicities in the association between physical environment and fruit and vegetable intake [23,25]. In the present study, interactions tested revealed that sex, age, annual income, and education did not moderate the association between perceived accessibility to healthy foods and diet quality. It would have been relevant to know if participants were the primary food shopper of their household since it could be expected that individuals who are in charge of grocery shopping are more influenced by or conscious of the food accessibility.

Strengths and limitations of this study deserve to be acknowledged. First, the exclusive use of validated tools that were specifically developed for the study population improves the reliability of the results obtained. Also, the use of an index of the overall diet quality brings novelty to this field of research where most studies to date have used specific proxies of healthy eating, such as intake of fruits and vegetables. Moreover, diet quality was measured based on three 24 h food recalls, increasing the likelihood of capturing participants’ usual intakes [68,69]. The fact that recruitment was performed using a random list of phone numbers is another strength of this study, allowing us to reach participants who do not usually volunteer to participate in such studies. Unfortunately, this recruitment method was not enough to prevent highly educated individuals to be overrepresented in our sample (45.8% having a university degree vs. 31% for the population of the Province of Québec [70]), thus limiting the generalizability of the results. Another limitation of this study is its cross-sectional design; therefore, it is not possible to know if improvement in the social or physical food environment would lead to improved diet quality.

## 5. Conclusions

In conclusion, the present study sheds light on the associations of social and perceived physical environment with overall diet quality among French-speaking adults of the Province of Québec. Consistent with previous observations [65], our results suggest that social environment, more precisely social support from close others at home, may have a stronger influence on healthy eating than perceived physical environmental factors. These findings support the added value of healthy eating promotion programs focusing on social support at home, especially for more socioeconomically disadvantaged individuals, whose efforts to eat healthily may be more easily thwarted by non-supportive households.

## Figures and Tables

**Table 1:** Sample characteristics.

**Characteristics**	*n* (%)
Female	471 (49.5)
**Age (years)**	
18–34	348 (36.6)
35–49	283 (29.7)
50–65	321 (33.7)
**Ethnicity**	
Caucasian	868 (91.2)
**Highest level of education**	
High school or less	216 (22.7)
College	290 (30.5)
University	436 (45.8)
Missing value or prefer not to answer	10 (1.1)
**Occupation**	
Worker	604 (63.5)
Retired	127 (13.3)
Student	114 (12.0)
No job	36 (3.8)
Other	49 (5.2)
Missing value or prefer not to answer	22 (2.3)
**Marital status**	
Married or living under common law	604 (63.4)
Other status	284 (29.8)
Missing value or prefer not to answer	64 (6.7)
**Living arrangement (living with…)**	
Partner only	243 (25.5)
Partner and children	367 (38.6)
Children only	55 (5.8)
Family member (other than a partner and children)	125 (13.1)
Roommate	22 (2.3)
Alone	127 (13.3)
Missing value or prefer not to answer	13 (1.4)
**Smoking status**	
Current smoker	120 (12.6)
Non-smoker or former smoker	830 (87.2)
Missing value	2 (0.2)

**Note**: *n* = 952 participants included for the analyses for the objective regarding social support for healthy eating.

**Table 2:** Scores for the four subscales of the Social Support for Healthy Eating questionnaire for the whole sample and according to sociodemographic characteristics.

	Supportive Actions at Home		Non-Supportive Actions at Home		Supportive Actions Outside of Home		Non-Supportive Actions Outside of Home	
	Mean ± SD	*p*	Mean ± SD	*p*	Mean ± SD	*p*	Mean ± SD	*p*
Whole sample	3.2 ± 0.9	*n*/a	1.6 ± 0.5	*n*/a	2.6 ± 0.8	*n*/a	1.6 ± 0.5	*n*/a
**Sex**		0.0343		0.0001		0.0008		0.79
Women	3.1 ± 0.9		1.7 ± 0.6		2.7 ± 0.8		1.6 ± 0.5	
Men	3.2 ± 0.9		1.6 ± 0.5		2.6 ± 0.8		1.6 ± 0.5	
**Age**		0.0179 *		<0.0001 *		0.0004 *		<0.0001 *
18–34 year	3.2 ± 0.9		1.7 ± 0.5		2.8 ± 0.7		1.8 ± 0.6	
35–49 year	3.2 ± 0.9		1.7 ± 0.5		2.7 ± 0.8		1.6 ± 0.4	
50–65 year	3.0 ± 1.0		1.5 ± 0.5		2.5 ± 0.8		1.6 ± 0.5	
**Education**		0.19 ^†^		0.11 ^†^		0.0405 ^†^		0.48 ^†^
High school or less	3.1 ± 0.9		1.7 ± 0.5		2.6 ± 0.9		1.7 ± 0.5	
College or university	3.2 ± 0.9		1.6 ± 0.5		2.7 ± 0.8		1.6 ± 0.5	
**Income**		0.0219 ^†^		0.49 ^†^		0.07 ^†^		0.20 ^†^
<low-income cut-off	3.0 ± 1.0		1.6 ± 0.6		2.6 ± 0.9		1.7 ± 0.7	
>low-income cut-off	3.2 ± 0.9		1.6 ± 0.5		2.7 ± 0.8		1.6 ± 0.5	
**Living with**		<0.0001 *		<0.0001 *		0.26 *		0.0124 *
Partner only	3.4 ^a^± 0.7		1.6 ^a^ ± 0.5		2.6 ± 0.8		1.6 ^a,b^ ± 0.5	
Partner and children	3.3 ^a,b^ ± 0.7		1.8 ^b^ ± 0.5		2.6 ± 0.8		1.6 ^c^ ± 0.5	
Children only	3.1 ^b,c^ ± 0.7		1.7 ^a,b^ ± 0.5		2.8 ± 0.9		1.7 ^a,b,c^ ± 0.6	
Family member	3.3 ^a,b,c^ ± 0.8		1.7 ^a,b^ ± 0.5		2.6 ± 0.8		1.8 ^b^ ± 0.6	
Roommate	3.0 ^c^ ± 0.8		1.6 ^a,b^ ± 0.5		2.8 ± 0.8		1.7 ^a,b,c^ ± 0.4	
Alone	2.0 ^d^ ± 1.1		1.2 ^c^ ± 0.3		2.6 ± 0.8		1.5 ^a,c^ ± 0.4	

**Note**. *n* = 952. Scores are on a maximum of 5 points. Higher scores for the supportive and the non-supportive scales mean a higher frequency of these types of action. Differences in social support scores between categories were tested using generalized linear models (GENMOD), with Tukey adjustment for multiple comparisons. ^a, b, c^ Categories with different superscripted letters are significantly different (*p* < 0.05). * Adjusted for sex. ^†^ Adjusted for sex and age.

**Table 3:** Regression analyses of Canadian Healthy Eating Index (C-HEI) score on social support variables.

	C-HEI Score	
	B	(95% CI)
**Independent variables**		
Supportive actions at home	1.50	(0.46, 2.54)
Non-supportive actions at home	−3.06	(−4.95, −1.18)
Supportive actions outside of home	0.71	(−0.46, 1.87)
Non-supportive actions outside of home	0.73	(−1.20, 2.65)
**Covariates**		
Sex (1 = female, 2 = male)	−5.62	(−7.45, −3.80)
Age groups (1 = 18–34 year, 2 = 35–49 year, 3 = 50–65 year)	0.87	(−0.18, 1.92)
Education (1 = high school or less, 2 = college or university)	2.05	(−0.04, 4.15)
Household annual income (1 = under low-income cut-off, 2 = over low-income cut-off)	1.19	(−1.46, 3.85)
Marital status (1 = other status, 2 = married or living in common-law)	−0.36	(−2.47, 1.74)
Smoking status (1 = non-smoker/former smoker, 2 = current smoker)	−6.93	(−9.47, −4.40)
Nutrition knowledge (continuous score from 0 to 100)	0.13	(0.06, 0.19)
Reporting status (1 = under-reporter, 2 = plausible reporter, 3 = over-reporter)	2.66	(0.82, 4.51)

**Note**: *n* = 952. B = Unstandardized beta.

**Table 4:** Regression analyses of Canadian Healthy Eating Index (C-HEI) score on social support variables, stratified by education levels.

	C-HEI Score			
	High School or Less *		College or University **	
	**B**	**(95% CI)**	**B**	**(95% CI)**
**Independent variables**				
Supportive actions at home	1.37	(−0.69, 3.44)	1.55	(0.32, 2.77)
Non-supportive actions at home	−6.09	(9.92, −2.25)	−2.24	(−4.42, −0.06)
Supportive actions outside of home	1.10	(−1.19, 3.38)	0.77	(−0.60, 2.13)
Non-supportive actions outside of home	0.90	(−3.33, 5.13)	0.55	(−1.66, 2.76)
**Covariates**				
Sex (1 = female, 2 = male)	−5.01	(−9.02, −1.00)	−5.82	(−7.89, −3.76)
Age groups (1 = 18–34 year, 2 = 35–49 year, 3 = 50–65 year)	1.37	(−0.80, 3.53)	0.84	(−0.37, 2.05)
Household annual income (1 = under low-income cut-off, 2 = over low-income cut-off)	1.18	(−3.38, 5.75)	1.39	(−1.90, 4.67)
Marital status (1 = other status, 2 = married or living in common-law)	−3.26	(−7.29, 0.78)	0.34	(−2.16, 2.85)
Smoking status (1 = non-smoker/former smoker, 2 = current smoker)	−5.20	(−9.69, −0.72)	−7.66	(−10.75, −4.57)
Nutrition knowledge (continuous score from 0 to 100)	0.30	(0.17, 0.43)	0.07	(−0.01, 0.14)
Reporting status (1 = under-reporter, 2 = plausible reporter, 3 = over-reporter)	2.21	(−1.05, −5.47)	2.94	(0.70, 5.19)

**Note**: * *n* = 218; ** *n* = 734. B = Unstandardized beta.

**Table 5:** Accessibility to healthy foods scores for the whole sample and according to sociodemographic characteristics.

	Accessibility to Healthy Foods	
	Mean ± SD	*p*
Whole sample	3.8 ± 0.5	*n*/a
**Sex**		0.61
Women	3.8 ± 0.5	
Men	3.8 ± 0.5	
**Age**		0.23 *
18–34 year	3.8 ± 0.6	
35–49 year	3.9 ± 0.5	
50–65 year	3.8 ± 0.5	
**Income**		0.0007 ^†^
<low-income cut-off	3.7 ± 0.6	
>low-income cut-off	3.9 ± 0.5	
**Education**		0.0027 ^†^
High school or less	3.7 ± 0.6	
College or university	3.9 ± 0.5	

**Note**. *n* = 1035. Scores are on a maximum of 5 points. Differences in accessibility to healthy foods between categories were tested using generalized linear models (GENMOD), with Tukey adjustment for multiple comparisons. * Adjusted for sex. ^†^ Adjusted for sex and age.

**Table 6:** Regression analyses of Canadian Healthy Eating Index (C-HEI) score on perceived food environment variables.

	C-HEI Score	
	B	(95% CI)
**Independent variables**		
Perceived accessibility to healthy foods	0.01	(−1.51, 1.53)
Travel time from home to the main retailer (by car; 1 = Less than 10 min, 2 = 10 min or more)	1.31	(−0.62, 3.24)
**Covariates**		
Sex (1 = female, 2 = male)	−5.50	(−7.22, −3.78)
Age groups (1 = 18–34 year, 2 = 35–49 year, 3 = 50–65 year)	0.68	(−0.32, 1.69)
Education (1 = high school or less, 2 = college or university)	2.21	(0.18, 4.25)
Household annual income (1 = under low-income cut-off, 2 = over low-income cut-off)	1.74	(−0.77, 4.24)
Marital status (1 = other status, 2 = married or living in common-law)	0.50	(−1.39, 2.39)
Smoking status (1 = non-smoker/former smoker, 2 = current smoker)	−6.71	(−9.14, −4.27)
Nutrition knowledge (continuous score from 0 to 100)	0.13	(0.06, 0.19)
Reporting status (1 = under-reporter, 2 = plausible reporter, 3 = over-reporter)	2.33	(0.54, 4.11)

**Note**: *n* = 1035. B = Unstandardized beta.

## Supplementary Materials

The following are available online at https://www.mdpi.com/2072-6643/11/12/3030/s1, Table S1: Description of the C-HEI scoring for adults, Table S2: Unadjusted Pearson’s correlations between C-HEI and subscales of the Social Support for Healthy, Table S3: Sample characteristics.
