# Supplementary material for: Social Support, but Not Perceived Food Environment, Is Associated with Diet Quality in French-Speaking Canadians from the PREDISE Study
Source: Nutrients. 2019 Dec 12;11(12):3030. doi: 10.3390/nu11123030 (PMC6950594; doi:10.3390/nu11123030)
Supplement: Supplementary file 1 [file nutrients-11-03030-s001.zip › Table S1.docx]

**Table S1** Description of the C-HEI scoring for adults.

| **Component** | **Range of scores** | **Scoring criteria** |
| --- | --- | --- |
| **Adequacy** | **0 to 60 points** |  |
| Total vegetables and fruit | 0 to 10 points | Minimum: 0  Maximum: 7 to 8 servings |
| Whole fruit | 0 to 5 points | Minimum: 0  Maximum: 1.5 to 1.7 servings (21% of recommendation for total vegetables and fruit) |
| Dark green and orange vegetables | 0 to 5 points | Minimum: 0  Maximum: 1.5 to 1.7 servings (21% of recommendation for total vegetables and fruit) |
| Total grain products | 0 to 5 points | Minimum: 0  Maximum: 6 to 8 servings |
| Whole grains | 0 to 5 points | Minimum: 0  Maximum: 3 to 4 servings (50% of recommendation for total grain products) |
| Milk and alternatives | 0 to 10 points | Minimum: 0  Maximum: 2 to 3 servings |
| Meat and alternatives | 0 to 10 points | Minimum: 0  Maximum: 2 to 3 servings (150 to 225 grams) |
| Unsaturated fats | 0 to 10 points | Minimum: 0  Maximum: 30 to 45 grams |
| **Moderation** | **0 to 40 points** |  |
| Saturated fats | 8 to 10 points  0 to 8 points | Minimum 7% to 10% of total energy intake  10% to maximum 15% of total energy intake |
| Sodium | 8 to 10 points  0 to 8 points | Adequate intake to tolerable upper intake level  Tolerable upper intake level to twice tolerable upper intake level |
| “Other food” | 0 to 20 points | Minimum: 5% or less of total energy intake  Maximum: 40% or more of total energy intake |

For adequacy components, 0 points for minimum, 5 or 10 points for maximum or more, and proportional for amounts between minimum and maximum.

For moderation components, 10 or 20 points for minimum or less, 0 points for maximum or more, and proportional for amounts between minimum and maximum.

**Reference**: Garriguet D. Diet quality in Canada. Ottawa: Statistics Canada, 2009.
