# Supplementary material for: Social Support, but Not Perceived Food Environment, Is Associated with Diet Quality in French-Speaking Canadians from the PREDISE Study
Source: Nutrients. 2019 Dec 12;11(12):3030. doi: 10.3390/nu11123030 (PMC6950594; doi:10.3390/nu11123030)
Supplement: Supplementary file 1 [file nutrients-11-03030-s001.zip › Table S2.docx]

**Table S2.** Sample characteristics

|  | n (%) |
| --- | --- |
| Female | 517 (50.0) |
| Age (years) 18-34 35-49 50-65 | 373 (36.0) 307 (29.7) 355 (34.3) |
| Ethnicity Caucasian | 938 (90.6) |
| Highest level of education High school or less College University Missing value or prefer not to answer | 243 (22.4)  319 (30.8)  461 (44.5)  12 (1.2) |
| Occupation Worker Retired Student No job Other Missing value or prefer not to answer | 658 (63.6)  140 (13.5)  117 (11.3)  40 (3.9)  56 (5.4)  24 (2.3) |
| Marital status Married or living under common law Other status Missing value or prefer not to answer | 654 (63.2)  309 (29.9)  72 (7.0) |
| Living arrangement (living with…) Partner only Partner and children Children only Family member (other than a partner and children) Roommate Alone Missing value or prefer not to answer | 268 (25.9)  393 (38.0)  58 (5.6)  133 (12.9)  20 (1.9)  148 (14.3)  15 (1.4) |
| Smoking status Current smoker Non-smoker or former smoker Missing value | 135 (13.0)  898 (86.8)  2 (0.2) |

**Note:** n=1035 participants included for the analyse for the objective regarding perceived food environment.
